# Use of Rapid Ascertainment Process for Institutional Deaths (RAPID) to identify pregnancy-related deaths in tertiary-care obstetric hospitals in three departments in Haiti

**DOI:** 10.1186/s12884-017-1329-1

**Published:** 2017-05-16

**Authors:** Andrew T. Boyd, Erin N. Hulland, Reynold Grand’Pierre, Floris Nesi, Patrice Honoré, Reginald Jean-Louis, Endang Handzel

**Affiliations:** 1 Epidemic Intelligence Service, Centers for Disease Control and Prevention, Atlanta, GA USA; 2 Division of Global Health Protection, Centers for Disease Control and Prevention, Atlanta, GA USA; 3 Haiti Ministry of Public Health and Population, Port-au-Prince, Haiti; 4 Jhpiego, Port-au-Prince, Haiti; 5 Division of Global Health Protection, Centers for Disease Control and Prevention, Port-au-Prince, Haiti

**Keywords:** Maternal mortality, Pregnancy-related deaths, Rapid Ascertainment Process for Institutional Deaths (RAPID), Haiti

## Abstract

**Background:**

Accurate assessment of maternal deaths is difficult in countries lacking standardized data sources for their review. As a first step to investigate suspected maternal deaths, WHO suggests surveillance of “pregnancy-related deaths”, defined as deaths of women while pregnant or within 42 days of termination of pregnancy, irrespective of cause. Rapid Ascertainment Process for Institutional Deaths (RAPID), a surveillance tool, retrospectively identifies pregnancy-related deaths occurring in health facilities that may be missed by routine surveillance to assess gaps in reporting these deaths.

**Methods:**

We used RAPID to review pregnancy-related deaths in six tertiary obstetric care facilities in three departments in Haiti. We reviewed registers and medical dossiers of deaths among women of reproductive age occurring in 2014 and 2015 from all wards, along with any additional available dossiers of deaths not appearing in registers, to capture pregnancy status, suspected cause of death, and timing of death in relation to the pregnancy. We used capture-recapture analyses to estimate the true number of in-hospital pregnancy-related deaths in these facilities.

**Results:**

Among 373 deaths of women of reproductive age, we found 111 pregnancy-related deaths, 25.2% more than were reported through routine surveillance, and 22.5% of which were misclassified as non-pregnancy-related. Hemorrhage (27.0%) and hypertensive disorders (18.0%) were the most common categories of suspected causes of death, and deaths after termination of pregnancy were statistically significantly more common than deaths during pregnancy or delivery. Data were missing at multiple levels: 210 deaths had an undetermined pregnancy status, 48.7% of pregnancy-related deaths lacked specific information about timing of death in relation to the pregnancy, and capture-recapture analyses in three hospitals suggested that approximately one-quarter of pregnancy-related deaths were not captured by RAPID or routine surveillance.

**Conclusions:**

Across six tertiary obstetric care facilities in Haiti, RAPID identified unreported pregnancy-related deaths, and showed that missing data was a widespread problem. RAPID is a useful tool to more completely identify facility-based pregnancy-related deaths, but its repeated use would require a concomitant effort to systematically improve documentation of clinical findings in medical records. Limitations of RAPID demonstrate the need to use it alongside other tools to more accurately measure and address maternal mortality.

## Background

Today, the desire to reduce the burden of maternal mortality, as outlined within the United Nations’ (UN) Sustainable Development Goal (SDG) 3 of ensuring healthy lives, is universal [1]. However, capturing maternal deaths and assigning causes of maternal deaths are not straightforward. Capturing maternal deaths requires intensified surveillance and the use of multiple data sources [2]. In low-income countries, accurate capture of maternal deaths is further complicated by a lack of registration and routine medical certification of cause of death, although recent efforts are underway to improve such systems. [3–6] Low-income countries often also lack standardized or universally reliable methods and data sources for measuring and monitoring change in maternal mortality.

In these settings, final classification of a death as maternal requires intensive investigation. To narrow the pool of deaths to be investigated as suspected maternal deaths, the World Health Organization (WHO) recommends the surveillance of “pregnancy-related deaths”, a concept included in the International Statistical Classification of Diseases, 10^th^ revision (ICD-10) [7]. A pregnancy-related death is defined as the death of a woman while pregnant or within 42 days of termination of pregnancy, irrespective of the cause of death [8]. This is a more sensitive definition than that of maternal deaths, since pregnancy-related deaths can include those deaths due to accidental or incidental causes. This definition still allows measurement of deaths related to pregnancy, even though they do not strictly conform to the standard definition of “maternal death”, in settings where accurate information about definitive causes of death based on medical certification or a formal review process is unavailable. Alternatively, when medical certification or a formal review process for death is available, capturing pregnancy-related deaths provides a pool of deaths that can then be submitted to full review to definitively categorize these deaths as maternal. In this paradigm, the term “maternal deaths” is reserved for only those deaths determined after formal review to be definitively maternal.

The recognition of pregnancy-related deaths as an inexact substitute for or precursor to maternal deaths means that routine surveillance of “maternal deaths”, as well as literature exploring such surveillance, in settings where formal review is not done more accurately captures “pregnancy-related deaths”. To facilitate comparison of findings between our analysis and other literature, we attempted to differentiate between “maternal deaths” that were definitive maternal deaths after review and “maternal deaths” applied as a more general term. For the purposes of this article, both in its literature review and results, “pregnancy-related deaths” are as defined above and having not undergone formal review. “Maternal deaths” will refer to only those deaths that were found definitively to be maternal deaths after review with other tools.

The lack of standardized data sources for accurate capture of pregnancy-related deaths often contributes to underreporting and misclassification of pregnancy-related deaths in low-income countries, including those occurring in health facilities [9]. Underestimation of these institutional pregnancy-related deaths in routine surveillance is posited to be on the order of one-half to two-thirds of the total, with a substantial proportion of that underestimation due to misclassification of indirect pregnancy-related deaths, that is, those due to non-obstetric causes exacerbated by pregnancy, as non-pregnancy-related [10].

In order to combat the specific problem of underreporting and misclassification of pregnancy-related deaths occurring in health facilities, the University of Aberdeen’s Initiative for Maternal Mortality Programme Assessment (Immpact) designed a tool to identify and collect unreported pregnancy-related deaths in health facilities. Named the Rapid Ascertainment Process for Institutional Deaths (RAPID), it retrospectively identifies in-facility deaths that may have been missed during routine reporting of pregnancy-related deaths occurring in facilities. Its use entails the review of health facility records for in-facility deaths among women of reproductive age, defined as deaths of women between and including the ages of 15 and 49, and identifying pregnancy-related deaths among this group.

RAPID differs from routine pregnancy-related death surveillance tools in three ways. First, it requires the review of all deaths of women of reproductive age, not only those designated as pregnancy-related in the medical registers. The review includes examination of not only the medical registers but also medical dossiers, or records, of pregnancy-related deaths and deaths in which pregnancy status is not clear. Second, it requires the review of registers from all wards where women of reproductive age may be admitted, rather than only those in maternity wards. Finally, it guides the categorization of pregnancy-related deaths by the timing of death in relation to the pregnancy: during pregnancy, during delivery, or within 42 days after termination of pregnancy. RAPID has previously been used in low-income countries to estimate underreporting of in-facility pregnancy-related deaths national health information system [11, 12], document timing of pregnancy-related deaths in relation to the pregnancy [12], and evaluate changes in numbers of pregnancy-related deaths before and after an intervention [13].

Maternal mortality remains a significant public health problem in Haiti, but because Haiti lacks a robust system for vital registration or for accurate ascertainment of cause of death, including a lack of widespread capacity to perform maternal death autopsy, the current vital statistic system is incapable of accurate surveillance of maternal deaths. In 2015, the maternal mortality ratio (MMR) in Haiti was 359 per 100,000 live births, the highest ratio in the Western Hemisphere [14]. While Haiti’s MMR declined by 43% from 1990 to 2010, it has since plateaued [15]. Additionally, MMR estimates in Haiti are actually based on routine reporting of pregnancy-related deaths, not definitive maternal deaths, and the accuracy of those estimates have not been validated since the Haiti national Demographic Health Survey of 2005–2006. Methods to improve accuracy of measurement of maternal mortality in Haiti, which includes correction of underreporting of maternal deaths, could focus on first reporting pregnancy-related deaths. Little is known about the degree of underreporting of pregnancy-related deaths in hospitals in Haiti, and thus the scope of the contribution of that underreporting to overall pregnancy-related mortality in Haiti is unknown. RAPID was successfully utilized as part of a Maternal Death Surveillance and Response (MDSR) pilot implementation in two tertiary care hospitals in 2013 and 2014, which has informed the staged implementation of MDSR in these hospitals, but the small sample size and type of data collected limited their use.

In February 2016, to more systematically quantify the degree of underreporting and misclassification of pregnancy-related deaths in hospitals in Haiti, the Haiti Ministry of Health, the US non-governmental organization Jhpiego, and the US Centers for Disease Control and Prevention (CDC) used RAPID to conduct a review of deaths among women of reproductive age during the years 2014 and 2015 in a selection of tertiary care hospitals. This study represents the comprehensive use of RAPID to identify in-hospital pregnancy-related deaths and the most common suspected causes of those deaths. Additionally, the use of RAPID was intended to identify any systematic gaps in hospital-based reporting of pregnancy-related deaths and opportunities to improve reporting in the context of a resource-limited system.

## Methods

Over two weeks in February 2016, five CDC and Jhpiego personnel visited six urban, referral tertiary care hospitals that receive support from the US Agency for International Development (USAID) within three departments in Haiti. These hospitals are the only tertiary care facilities able to provide comprehensive emergency obstetric care in these departments, and they serve as referral hospitals for these services for their respective departments. None of these hospitals participated in the 2013–2014 pilot of MDSR in Haiti, and none has a protocol in place for conducting in-hospital mortality reviews. None of these hospitals has a protocol for capturing community deaths, and although cases of women of reproductive age who are dead on arrival to the hospital are recorded, minimal clinical information, including pregnancy status, is collected. Of the six hospitals, two each were in Ouest, Nord-Est, and Artibonite departments.

In accordance with RAPID methodology, and using the data forms of the RAPID tool to abstract data, study personnel reviewed patient registers in each hospital for 2014 and 2015 for all wards admitting women of reproductive age, defined as women aged 15–49 years [16]. The wards included the emergency department, maternity ward, obstetrics ward (in one hospital that had an obstetrics ward separate from the maternity ward), internal medicine ward, and operating theatre. Data were captured using the RAPID register data capture form in Microsoft Excel (Microsoft Office 2013), and collected variables included pregnancy status, date of delivery if it had occurred, and date of death. Thereafter, the medical dossiers, or hospital-based patient records, of those identified deaths, if available, were reviewed to confirm pregnancy status and clinical history. For this step, data were captured using the RAPID dossier data capture form in Excel, and collected variables included timing of death in relation to the pregnancy, pregnancy complications, and suspected cause of death (assignment of final cause of death requires the use of methodology other than RAPID). Documenting timing of death in relation to the pregnancy using the dossiers, rather than just through registers as prescribed by RAPID, was required because of poor documentation of this information in the registers. Additionally, because dossiers of deaths initially found in the register were often not retrievable, staff then reviewed available dossiers of all institutional deaths from the same time period to identify from them any additional deaths among women of reproductive age not captured in the registers. This more expansive review was performed at three of the six hospitals; at the other three, this process was not feasible. This last step was a slight modification of RAPID methodology, which prescribes the review of only those dossiers of deaths of women of reproductive age identified through register review.

**Fig. 1:**
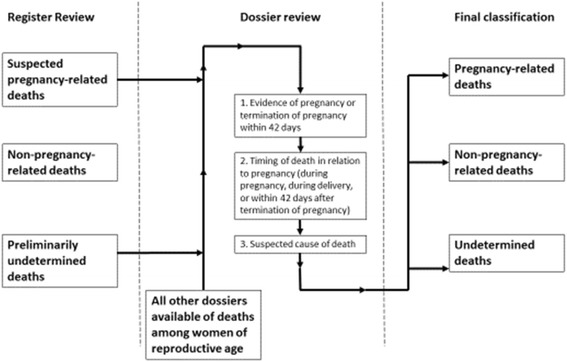
Process of identifying pregnancy-related deaths in tertiary-care obstetric hospitals in three departments in Haiti using RAPID

The procedures for categorizing a death of a woman of reproductive age as pregnancy-related or not, and then for capturing suspected cause of death, are displayed in Fig. 1. To determine a death as pregnancy-related or not, documentation of pregnancy or termination of pregnancy within 42 days of death was sought first in the register. If no pregnancy status was explicitly documented in the register, the death was considered preliminarily undetermined. For each preliminarily undetermined death, the dossier was then reviewed for documentation of evidence of pregnancy or termination of pregnancy within 42 days. If no pregnancy status was explicitly documented in the dossier, the death was finally categorized as undetermined. Among all deaths categorized as suspected pregnancy-related after register review, the dossier was examined for timing of death in relation to the pregnancy and for suspected cause of death, where available. If there was documentation of the death occurring during pregnancy, delivery, or within 42 days after termination of pregnancy, the death was given a final categorization of pregnancy-related. Among those deaths categorized as pregnancy-related, the specific timing of the death in relation to the pregnancy (during pregnancy, during delivery, or within 42 days of termination of pregnancy), if available, was recorded. If the suspected cause of death was explicitly mentioned in the dossier, that cause was recorded as the suspected cause of death. If the suspected cause of death was not explicitly mentioned, physicians among the study team assessed the constellation of signs, symptoms, and clinical course within the dossier to infer the most likely suspected cause of death. In cases where no or limited suspected cause of death information was available in the register or dossier, suspected cause of death was classified as unknown. These same procedures were followed for all deaths of women of reproductive age for which the dossiers were available for review. Pregnancy-related deaths found in a hospital ward without also being found in the maternity ward (henceforth to be used as a general term, inclusive of obstetric ward) were recorded as misclassified.

Data analysis was performed in SAS version 9.3 [17]. For each hospital and for each year, the total number of pregnancy-related deaths identified through RAPID was compared with the total number of pregnancy-related deaths reported by the hospital to routine surveillance systems: the Haiti National Monitoring, Evaluation, and Surveillance Interface (MESI) database for five of the six hospitals, and to a non-governmental organization database in one hospital. Next, to assess the degree of misclassification, the number of misclassified deaths, or those pregnancy-related deaths found in wards other than the maternity ward, was compared with total pregnancy-related deaths, inclusive of both correctly classified and misclassified deaths, by facility and by year. Identified pregnancy-related deaths were grouped by WHO Application of ICD-10 categories of cause of pregnancy-related deaths [7], and by whether they appeared in the maternity ward or in any other ward (ie, misclassified deaths). To examine whether suspected cause of death differed significantly based on ward of admission, Chi-square and Fisher’s exact tests were used to compare each category of cause of death versus all other categories combined. Due to a high number of unknown suspected causes of death, sensitivity analyses were performed wherein all individuals without a known cause of death were excluded from analysis. Next, Chi-square goodness-of-fit tests were run on the frequencies of categories of timing of pregnancy-related deaths, to assess whether the distribution of the timing of pregnancy-related deaths differed significantly from a uniform distribution. These tests were done both overall and for pairwise comparisons between the category “within 42 days after termination of pregnancy” and both other categories of the timing of pregnancy-related deaths. Finally, to determine the degree to which deaths among women of reproductive age were missing from both the RAPID register data capture form and the dossier data capture form, two-source capture-recapture methods were used to estimate the true number of in-hospital pregnancy-related deaths, using the Chapman estimator process [18]. The degree of overlap for the two sources was assessed, assuming a closed population, independence of sources, and equal probability of capture by either source for every woman of reproductive age in the population.

## Results

Register review identified 306 total deaths of women of reproductive age, of which 92 were initially identified as suspected pregnancy-related and four as non-pregnancy-related. Dossier review was then performed on the 210 deaths that remained undetermined as pregnancy-related or not after register review. Of those 210 deaths, dossier review found one that was pregnancy-related, one that was non-pregnancy-related, 40 that remained undetermined due to lack of pregnancy status information in the dossiers, and 168 that were missing dossiers, thereby providing no further information. As the registers did not provide comprehensive information on timing of death in relation to pregnancy, dossier review was required to further classify the 92 suspected pregnancy-related deaths as definitively pregnancy-related or non-pregnancy related. Of the 92 suspected pregnancy-related deaths located through register review, 88 were finally categorized as pregnancy-related, while four were non-pregnancy-related. Thereafter, upon review of all available dossiers of all in-hospital deaths, 67 additional deaths of women of reproductive age were identified, of which 22 were pregnancy-related, 43 were non-pregnancy-related, and two remained undetermined. Of note, only three of the six hospitals had available dossiers of all in-hospital deaths for review, and one hospital‘s (Hospital 6) stored dossiers were not accessible for review at all. Overall, there were 373 deaths of women of reproductive age identified, of which 111 were pregnancy-related, 52 were definitively non-pregnancy-related, and 210 were undetermined. Finally, among the 92 pregnancy-related deaths captured through register review, 22 (23.9%) had dossiers available for secondary review.

The 111 pregnancy-related deaths identified across all six hospitals and across both years was 25.2% more than were reported through routine surveillance (Table 1). Four of the six hospitals had underreporting of at least 33% for one of the two years reviewed, and three of those had at least one year with over 48% underreporting. In contrast, in Hospital 3, RAPID identified only 56% of the pregnancy-related deaths reported through routine surveillance. Each selected hospital had misclassified deaths in at least one of the two years, and overall, 22.5% (25/111) of all identified pregnancy-related deaths were misclassified (Table 1).

**Table 1:** Total numbers of pregnancy-related deaths identified, reported, and misclassified, and total numbers of undetermined deaths among women of reproductive age, by selected hospital and year, 2014-2015

Institution	Year	Total number of pregnancy-related deaths identified	Number of pregnancy-related deaths reported^b^	Percent difference between pregnancy-related deaths identified and reported (%)	Number of misclassified pregnancy-related deaths	Percent misclassification (%)	Number of undetermined deaths
Hospital 1^a^	2014	10	5	50.0	2	20.0	41
	2015	13	6	53.8	0	0	29
Hospital 2	2014	10	8	20.0	3	30.0	3
	2015	14	5	64.3	7	50.0	0
Hospital 3^a^	2014	6	7	−16.7	3	50.0	16
	2015	4	11	−175.0	4	100.0	7
Hospital 4	2014	4	6	−50.0	1	25.0	10
	2015	4	4	0	0	0	11
Hospital 5	2014	25	13	48.0	2	8.0	41
	2015	18	16	11.1	1	5.6	30
Hospital 6^a^	2014	3	2	33.3	2	66.7	13
	2015	0	0	0	0	0	9
Totals		111	83	25.2	25	22.5	210

^a^Not all dossiers of all women of reproductive age were available for review at these institutions

^b^Source is MESI for five of six hospitals and a non-government organization database for one hospital

The WHO Application of ICD-10 categories of suspected causes of identified pregnancy-related deaths showed that, across all hospitals for both years, the most common categories of pregnancy-related death were “unknown” (31.5%, 35/111), hemorrhage (27.0%, 30/111), and hypertensive disorders (18.0%, 20/111) (Table 2). Chi-square and Fisher’s exact tests of proportions showed that only “Group 4-Pregnancy-related infection”, when compared to all other categories, showed a statistically significant difference by ward of admission, with pregnancy-related infections over four times more likely to be found in registers or dossiers of wards other than the maternity ward. However, after performing sensitivity analyses to exclude unknown causes of pregnancy-related deaths, this association was no longer statistically significant. None of the other sequential tests showed significant differences in suspected cause of death between those in the maternity ward and those in other wards, although it is notable that there was an observed two-fold higher frequency of indirect pregnancy-related cause of death (inclusive of HIV/AIDS, chronic liver disease, and stroke) for those seen in other wards versus the maternity ward despite non-significance.

**Table 2:** Category of suspected cause of pregnancy-related death across all hospitals and both years, stratified by those identified in the maternity ward and those identified in all other wards

WHO Application of ICD10 category of suspected cause of pregnancy-related death	Total *N* = 111	Maternity Ward *N* = 86	Other Ward *N* = 25	*P*-value^a^
Group 1-Pregnancy with abortive outcome	3 (2.7%)	2 (2.3%)	1 (4.0%)	0.539
Group 2-Hypertensive disorders in pregnancy	20 (18.0%)	17 (19.8%)	3 (12.0%)	0.556
Group 3-Obstetric hemorrhage	30 (27.0%)	24 (27.9%)	6 (24.0%)	0.802
Group 4-Pregnancy-related infection	7 (6.3%)	3 (3.5%)	4 (16.0%)	*0.044* ^b^
Group 5-Other obstetric complications (e.g., venous complications, obstetric embolism, obstetric trauma)	8 (7.2%)	6 (7.0%)	2 (8.0%)	1.000
Group 6-Complications of anesthesia	1 (0.9%)	0 (0.0%)	1 (4.0%)	0.225
Group 7-Indirect pregnancy-related (i.e., systemic diseases complicated by pregnancy)	11 (9.9%)	5 (5.8%)	3 (12.0%)	0.376
Group 8-Unknown (i.e., pregnancy-related death with unspecified cause)	35 (31.5%)	29 (33.7%)	5 (20.0%)	0.226

^a^ *P*-value calculated via Fisher’s exact test comparing each cause separately compared with all other causes combined

^b^﻿*P*-value less than 0.05

Analysis of the timing of pregnancy-related deaths showed that 37.8% (42/111) were within 42 days after termination of pregnancy, a higher proportion than those dying while pregnant or during delivery (Table 3). Among the 42 pregnancy-related deaths that were identified as occurring “within 42 days of termination of pregnancy”, 25 (59.5%) appeared in the ward registers. Chi-square goodness-of-fit tests revealed that the frequencies of categories of timing of pregnancy-related deaths deviated significantly from a uniform distribution, indicating that deaths within the various categories of timing of death did not occur with equal frequency. Furthermore, sequential pairwise comparisons between the category of “death within 42 days of termination of pregnancy” and the other two specific categories for timing revealed that the frequency of death within 42 days of pregnancy termination differed significantly from the expected proportion of 50% of deaths in each of the two compared categories. Of note, 48.7% (54/111) of pregnancy-related deaths could not be classified precisely as occurring during pregnancy, during delivery, or within 42 days of termination of pregnancy.

**Table 3:** RAPID categories of timing of death among all 111 pregnancy-related deaths

Timing of death in relation to pregnancy (among pregnancy-related deaths)^a^	Total (%)
Died while pregnant	8 (7.2%)
Died during delivery	7 (6.3%)
Died within 42 days of termination of pregnancy	42 (37.8)
Specific timing of death in relation to pregnancy not specified	54 (48.7%)

^a^Hospital 6 had no dossiers available for review

In three hospitals, use of capture-recapture methodology demonstrated that an estimated 27.3%, 25.0%, and 23.9% of pregnancy-related deaths, respectively, were not captured by either data source (Table 4). Of note, this was only performed for the three hospitals in which dossiers of institutional deaths were largely available to allow completion of the dossier data capture form.

**Table 4:** Results of capture-recapture methodology of pregnancy-related deaths at three hospitals

Institution	Group	Deaths captured by register data capture form	Deaths captured by dossier data capture form	Deaths captured by both sources	Estimated number of deaths missing from both sources	Estimated total number of deaths	Percentage of missing deaths (95% CI)
Hospital 2	Pregnancy-related deaths	16	15	7	9	33	27.3% (20.3–41.7)
Hospital 4	Pregnancy-related deaths	6	4	2	3	11	25.0% (16.5–51.3)
Hospital 5	Pregnancy-related deaths	34	22	13	14	57	23.9% (19.1–31.8)

## Discussion

This analysis contributes to the literature by using a comprehensive tool to retrospectively examine deaths of women of reproductive age to identify and describe pregnancy-related deaths in six tertiary care facilities providing obstetric and neonatal care in three departments in Haiti, a low-income country with a high maternal mortality ratio.

In comparing the pregnancy-related deaths identified through RAPID with pregnancy-related deaths reported to routine surveillance systems, the degree of underreporting across the six hospitals varied widely. Four of the six hospitals had underreporting of at least 33% for one of the two years reviewed. However, this pattern of underreporting was not universally seen; at Hospital 3, for instance, the use of the RAPID tool identified just over half of the pregnancy-related deaths reported. In this case, use of RAPID to identify deaths was hampered by the fact that registers from other wards were not uniformly available for review. In addition, though Hospital 3 centrally stored its dossiers of in-hospital deaths by the month, nine months’ worth of dossiers of deaths during the time period of interest were missing. Both of these facts prevented the identification of deaths with RAPID that may have been reported to the routine surveillance system. There was insufficient information about the missing dossiers to be able to say if they were systematically different from those found, whether in ward of death or timing of death in relation to a pregnancy. Across all six hospitals and both years, RAPID identified about 25% more pregnancy-related deaths than were reported through routine surveillance. This underreporting is less than that found in Tanzania, where RAPID identified 53% more pregnancy-related deaths over five years than were reported to the national health information system [11]. It is closer to that found in a study of hospitals in eight districts in Namibia over three years, in which RAPID identified 39% more pregnancy-related deaths than routine surveillance had identified [12]. A study in Indonesia used RAPID in two district hospitals over two years to identify pregnancy-related deaths, followed by a full review, to find a number of maternal deaths 2.3 times higher than the number of reported pregnancy-related deaths [10]. A reproductive age mortality survey done in five hospitals in Surinam covering the years 1981 to 1990 identified 1.3 times the number of maternal deaths than were reported for the entire country, despite the unavailability of 36% of dossiers for review [9]. Underreporting of maternal deaths is not limited to low- and middle-income countries, with studies using investigatory tools other than RAPID finding varying degrees of underreporting in the US, Finland, France [19], Austria [20], and the Netherlands [21].

Another common finding in this analysis was the misclassification of pregnancy-related deaths as non-pregnancy-related deaths. About 23% of all identified pregnancy-related deaths were misclassified. Pregnancy-related deaths categorized as pregnancy-related infections were statistically more likely to be found in wards other than the maternity ward compared with all other categories of pregnancy-related deaths, though the significance did not remain when analysis was limited to only those deaths with a documented likely cause. The misclassification of pregnancy-related infections has not been shown in other literature. The observed two-fold higher frequency of indirect pregnancy-related cause of death for those seen in other wards versus the maternity ward was not statistically significant, but the finding of misclassified indirect pregnancy-related deaths was demonstrated in a previous study in Indonesia [10]. In Haiti, where routine counting and reporting of pregnancy-related deaths are done through register review by public health officers, it may be more likely that deaths instead recorded in dossiers or in other wards’ registers are missed.

RAPID cannot provide a definitive cause of death; assignment of a final cause of death requires the use of tools other than RAPID. That said, it can provide a suspected cause of death based on information found on dossier review. In fact, among 111 identified pregnancy-related deaths in this study, all but five either appeared in the maternity register (and thus were pregnancy-related) or had a discernible suspected cause. Examination of the categories of suspected cause of death also showed patterns consistent with other studies’ findings. Aside from the most common category of pregnancy-related death, “unknown”, which was largely due to missing or insufficient information in dossiers, the two most common causes of pregnancy-related deaths, inclusive of misclassified pregnancy-related deaths, were hemorrhage and hypertensive disorders, including preeclampsia/eclampsia. Similarly, a pooled analysis of ten datasets of maternal deaths in Latin America and the Caribbean found hypertensive disorders (26%) and hemorrhage (21%) the most common categories of cause of death [22]. Chronic hypertension was identified as a risk factor for development of preeclampsia/eclampsia in an analysis of a health facility-based study across 24 low- and middle-income countries [23]. Thus, the findings that a significant proportion of identified pregnancy-related deaths were due to hypertensive disorders suggests that more extensive screening and treatment of elevated blood pressure in antenatal care may be appropriate.

Analysis of the timing of pregnancy-related deaths showed a statistically significant higher proportion of deaths occurring within 42 days after termination of pregnancy than occurred during pregnancy or during delivery. The postpartum period, especially the first 24 h after giving birth, is the time when death is most likely to occur, mainly due to hemorrhage or sepsis [24]. In Haiti, where only 67% of women have sufficient health care access to receive the recommended four antenatal care visits [25], those who die in the postpartum period may be those who delay seeking care or may lack access to care for postpartum complications, making death more likely once they arrive in the hospital.

It is likely that this method missed pregnancy-related deaths at several different levels. Even after the completion of RAPID, designed to document deaths as pregnancy-related or not, and with the additional step of review of all in-hospital deaths to find additional information on deaths among women of reproductive age, 210 deaths remained with an undetermined pregnancy status, as a result of a combination of missing registers, missing dossiers, or inadequate information about pregnancy status or clinical presentation in available dossiers. Part of the utility of RAPID is that review of dossiers provides supplemental information about pregnancy status and timing of death in relation to the pregnancy, but in three hospitals visited, dossiers of deaths with undetermined pregnancy status were either not available or largely unavailable for review. In comparing findings of numbers of misclassified pregnancy-related deaths and number of undetermined deaths between hospitals with largely available dossiers and hospitals without them, there was no obvious pattern of misclassification or in number of undetermined deaths. Additionally, the dossiers of pregnancy-related deaths that were available often did not provide timing in relation to pregnancy: timing information was not available for nearly half of pregnancy-related deaths. These findings reflect the fact that across these hospitals, though standardized registers and dossiers exist, they are not always used; moreover, the quality and completeness of data in registers and dossiers is not routinely reviewed. Thus, it seems that in this study, having dossiers available for review may have found additional deaths, but dossiers were not uniformly helpful in providing supplemental information on pregnancy status or on timing of death in relation to pregnancy. A proportion of those deaths that remained with undetermined pregnancy status are likely true pregnancy-related deaths.

Additionally, the use of RAPID pointed out some pervasive problems with storage and documentation of medical information in these facilities, suggesting that some changes in practices and management of medical records are needed. In hospitals that did not store dossiers of in-hospital deaths, it was difficult to assure that all deaths of women of reproductive age were reviewed. Thus, the study points out the need for hospitals to systematically and separately document in-hospital deaths, perhaps in a death register. In addition to missing information about pregnancy status and timing of death in relation to a pregnancy in reviewed dossiers, the cause of pregnancy-related deaths was often not present. Systematic inclusion of pregnancy history and how long ago delivery was, as well as documentation of death through use of death certificates, would enhance the usefulness of medical dossiers to capture pregnancy status, timing of death, and assignment of cause of death, thus making the findings of RAPID more complete. Such changes would require training for clinicians in ICD-10 categorization of pregnancy-related deaths and in the use of death certificates. Other improvements in reporting pregnancy-related deaths could come from a complementary qualitative review conducted among health care workers.

In addition to this high proportion of deaths of woman of reproductive age with undetermined pregnancy status, imprecise timing of death, or unknown cause of death, capture-recapture methods demonstrated that across three hospitals, approximately one-quarter of in-facility pregnancy-related deaths were missed. The use of capture-recapture analysis here may be limited further in that it is unknown to what degree the assumptions surrounding capture-recapture were met. It is unlikely that the population remained entirely stable throughout the study period, and it was not possible to assess whether every woman of reproductive age had the same ability to be captured by either list. Furthermore, the two sources used may not be entirely independent. It is also worth noting that capture-recapture could only be performed in three of the six hospitals, due to the lack of largely available dossiers in the other three hospitals, or due to complete dependency between the dossiers and the registers.

Finally, though RAPID identifies hospital-based pregnancy-related deaths, only 36% of Haitian pregnant women deliver in a facility [25]. Where women lack access to high-quality medical care, pregnancy-related deaths may not come to the attention of the medical system at all. Thus, while this analysis showed underreporting of in-hospital pregnancy-related mortality, Haiti’s overall pregnancy-related mortality ratio is likely even higher. A more comprehensive view of this overall pregnancy-related mortality would require tools other than RAPID.

Limitations of this study were as follows. The numbers of identified pregnancy-related deaths found through use of RAPID were compared directly with aggregated numbers reported to routine surveillance system, but if routine surveillance does not systematically capture deaths that occur after delivery and up to 42 days after termination of pregnancy, then the comparison may not be exact. Furthermore, disaggregated or identifying data on deaths reported to routine surveillance systems were not available, and thus we cannot comment on the possibility of duplication of deaths captured by routine surveillance system, or whether certain causes or timings of death were more likely to be captured by the routine system. Discovering if these problems exist in routine reporting of pregnancy-related deaths would require a data quality review of routine reporting, which was beyond the scope of this study. Third, the small sample sizes in the comparison of cause of death by ward of registration limited the power to detect significant differences between misclassified and properly classified deaths. Finally, reviewed facilities were tertiary care facilities able to provide comprehensive emergency obstetric care in these three departments, and thus may have a different case mix or mortality rate than other facilities in Haiti. This difference limits the representativeness of the findings.

## Conclusions

This analysis used a data abstraction tool, RAPID, that had previously been used in other low-income countries, to more comprehensively document health facility-based pregnancy-related mortality in selected tertiary care hospitals providing comprehensive emergency obstetric care in Haiti. The analysis represents the first time, outside of a small pilot study, it has been used in the country, and it is an attempt to provide a first step toward understanding gaps in measuring maternal mortality in Haiti. RAPID demonstrated both underreporting and misclassification of pregnancy-related deaths across the facilities, as well as a widespread problem of missing data.

RAPID could be used periodically as a standard tool to monitor routine facility-based surveillance of pregnancy-related deaths, specifically by enhancing the comprehensiveness of capture of pregnancy-related deaths in hospitals, since it emphasizes review of other ward registers and dossiers of deaths among women with an undetermined pregnancy status in the register. Its ongoing use could also help monitor improvement in reporting, and importantly, provide a description of location and timing of facility-based pregnancy-related deaths in Haiti. In this way, adequate and targeted resources could be directed at reducing mortality in this vulnerable population. Such repeated use, though, would require a concomitant effort to systematically include pregnancy status in dossiers and to maintain a system for localizing the dossiers.

Finally, RAPID is a tool to find pregnancy-related deaths, and its use highlights the new distinction in the ICD-10 between pregnancy-related deaths and definitive maternal deaths. Confirming collected pregnancy-related deaths as maternal deaths requires more intensive investigation using other tools. RAPID is meant to provide a first step in capturing maternal deaths. RAPID, then, could be used by public health practitioners and administrators in Haiti and countries like it as one tool, alongside several others in a framework such as a reproductive age mortality study (RAMOS), in order to more comprehensively and accurately measure, understand, and address maternal mortality in Haiti and other low-income countries.

## Abbreviations

CDC: U.S. Centers for Disease Control and Prevention
ICD-10: International Statistical Classification of Diseases, 10^th^ revision
ICD-10MM: International Statistical Classification of Diseases, 10^th^ revision, Application to Maternal Mortality
Immpact: Initiative for Maternal Mortality Programme Assessment
MDSR: Maternal Death Surveillance and Response
MESI: Haiti Monitoring, Evaluation, and Surveillance Interface
MMR: Maternal mortality ratio
RAMOS: Reproductive age mortality study
RAPID: Rapid Ascertainment Process for Institutional Deaths
SDG: Sustainable Development Goal
UN: United Nations
USAID: U.S. Agency for International Development
WHO: World Health Organization
